# Facilitators and barriers to engaging with the DREAMS initiative among young women who sell sex aged 18–24 in Zimbabwe: a qualitative study

**DOI:** 10.1186/s12905-023-02374-4

**Published:** 2023-05-12

**Authors:** Fortunate Machingura, Joanna Busza, Gracious Madimutsa Jamali, Memory Makamba, Phillis Mushati, Tarisai Chiyaka, James Hargreaves, Bernadette Hensen, Isolde Birdthistle, Frances Mary Cowan

**Affiliations:** 1 Centre for Sexual Health and HIV/AIDS Research, (CeSHHAR) Zimbabwe, 4 Bath Road, Belgravia, Harare, Zimbabwe; 2 Department of International Public Health, Liverpool School of Tropical Medicine, Liverpool, UK; 3 Centre for Evaluation, London School of Hygiene and Tropical Medicine, London, UK; 4 Department of Clinical Research, London School of Hygiene & Tropical Medicine, London, UK; 5 Department of Population Health, London School of Hygiene & Tropical Medicine, London, UK

**Keywords:** Zimbabwe, Qualitative Research, Young Women, Vulnerability, Social Intervention

## Abstract

**Background:**

Adolescent girls and young women (AGYW) are at high risk of contracting HIV and exchanging sex for financial or material support heightens their risk. In Zimbabwe, the DREAMS initiative integrated education and employment opportunities within HIV health promotion and clinical services for vulnerable young women, including those who sell sex. While most participants accessed health services, fewer than 10% participated in any social programmes.

**Methods:**

We conducted semi-structured qualitative interviews with 43 young women aged 18–24 to understand their experiences of engaging with the DREAMS programme. We purposively sampled participants for diversity in level of education, type and location of selling sex. We analysed the data by applying the Theoretical Domains Framework to explore facilitators and barriers to engaging with DREAMS.

**Results:**

Eligible women were motivated by hopes of escaping poverty, and their longer-term engagement was sustained through exposure to new social networks, including friendships with less vulnerable peers. Barriers included opportunity costs and expenses such as transport or equipment required for job placements. Participants also described pervasive stigma and discrimination related to their involvement in selling sex. Interviews highlighted the young women’s struggles in a context of entrenched social and material deprivation and structural discrimination that hindered their ability to take up most of the social services offered.

**Conclusions:**

This study demonstrates that while poverty was a key driver of participation in an integrated package of support, it also constrained the ability of highly vulnerable young women to benefit fully from the DREAMS initiative. Multi-layered HIV prevention approaches such as DREAMS that seek to alter complex and longstanding social and economic deprivation address many of the challenges faced by YWSS but will only succeed if the underlying drivers of HIV risk among YWSS are also addressed.

**Supplementary Information:**

The online version contains supplementary material available at 10.1186/s12905-023-02374-4.

## Contributions to the literature

- The DREAMS initiative that was implemented in Zimbabwe integrated education and employment opportunities within HIV health promotion and clinical services for vulnerable young women, including YWSS
- While there is widespread acceptance that AGYW’s vulnerability needs to be addressed holistically, in reality this is extremely challenging, particularly for YWSS
- Community mobilisation activities are critical to developing strong relationships, networks, and collective strategies for overcoming social barriers
- This study identified facilitators and barriers to participation in DREAMS among YWSS in Zimbabwe, which can help improve programmatic approaches to reduce HIV incidence among YWSS

## Background

In sub-Saharan Africa, adolescent girls and young women (AGYW) aged 15–24 years are at high risk of contracting HIV, accounting for approximately one quarter of new infections despite comprising only 10% of the population [1]. In eastern and southern Africa, 30% of new HIV infections in 2019 occurred in AGYW [2]. Exchanging sex for money or other material support heightens this risk, and young women who sell sex (YWSS) aged < 25 have been identified as especially vulnerable [3]. In Zimbabwe, programme data collected by the national HIV programme for sex workers, *Sisters with a Voice*, shows that HIV prevalence among YWSS rises steeply between ages of 18 and 24 years from 5% to over 50%, respectively [4].

The inequitable burden of HIV among YWSS is driven by intersecting biological, behavioural and structural factors [5, 6]. Social drivers of HIV among YWSS in Zimbabwe include gendered inequities in education and employment opportunities, behavioural norms supporting men’s multiple partnerships and age disparities within relationships, and intimate partner violence [4] [7, 8]. While selling sex is one of few ways for young women to earn an income, YWSS struggle to negotiate condom use and are less likely to use health services for fear of stigma and discrimination compared to older sex workers, exacerbating their risk of HIV acquisition [8–10].

The DREAMS Partnership (“Determined, Resilient, Empowered, AIDS-free, Mentored, and Safe”) was devised to address the risk environment leading to girls’ and young women’s vulnerability to HIV in ten sub-Saharan African countries [11]. DREAMS introduced a multisectoral package of combination HIV prevention to reduce HIV incidence among AGYW aged 15–24 years, including YWSS as those at highest risk. In Zimbabwe, six DREAMS implementing partners provided support for YWSS to attend school or vocational training alongside health promotion and clinical services for HIV and STIs in six districts, integrated into the existing *Sisters with a Voice* (Sisters) targeted programme for sex workers. In four districts, Sisters also referred YWSS aged 18–24 years for pre-exposure prophylaxis (PrEP) in accordance with national guidelines at the time [12].

To evaluate DREAMS in Zimbabwe, HIV incidence was compared between a cohort of YWSS in 2 districts that received DREAMS activities and a cohort of YWSS in 4 districts that did not [12, 13]. The impact evaluation found HIV incidence to be lower in DREAMS districts, at 3.1/100 person-years compared to 5.3/100 person years in non-DREAMS districts [14]. While many YWSS took up clinical services through DREAMS, fewer engaged with its social and economic opportunities. Among 652 YWSS surveyed in DREAMS districts, 59 (9%) received any social interventions in the previous 12 months. Of these, 26 (4%) had received cash transfers or educational subsidies, 13 (2%) had participated in a continuing education programme, 13 (2%) participated in job placements and 33 (5%) had enrolled in an internal savings and lending group. Yet 96% used HIV-related clinical services including attending Sister’s clinics. Of 538 surveyed YWSS who tested HIV negative at enrolment about 47% had ever been offered PrEP, 28% ever took-up PrEP and 12% continued on PrEP [14]. Thus, despite efforts to address YWSS’ multiple and intersecting vulnerabilities, DREAMS achieved greater coverage of health service provision than social services.

This paper presents qualitative data collected from YWSS attending Sisters clinical services who were eligible for referral to DREAMS social protection activities. We aimed to understand why YWSS did or did not start and continue to participate in programme activities. We explored their perceptions and experiences of the programme over 2 years outlining the barriers and facilitators to the DREAMS initiative.

## Methods

We collected data from YWSS aged 18–24 residing in two Zimbabwean cities selected for DREAMS interventions. Transgender young women were not included in this study because the DREAMS intervention targeted AGYW and transwomen were not part of the target population. Also, in 2017 when the DREAMS intervention started, the Sisters’ Programme had not yet extended services to transgender women. However, this service has since been extended to the trans community since September 2021. In each city, Sisters offered HIV testing, condom promotion and distribution, community mobilisation and referral to legal advice [15]. YWSS were offered referrals to other DREAMS implementing partners offering oral PrEP and social protection. Social protection included: funds and support for secondary school and vocational education, Internal Savings and Lending (ISAL) schemes, and job placement training (e.g. events management, catering, tourism, vehicle mechanics) [12].

**Fig. 1:**
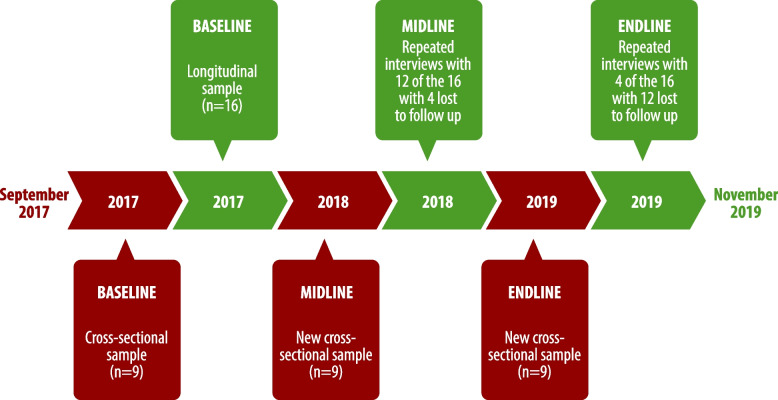
Study Design

This qualitative study was conducted in two predominantly urban district sites that referred YWSS into the DREAMS programme (Table 1). We collected data between September 2017 and November 2019. We conducted semi-structured interviews with 43 YWSS purposively selected for diversity in level of education, type and location of selling sex. We collected both longitudinal and cross-sectional data to capture different experiences. We planned to follow 25 DREAMS participants throughout the intervention period, with interviews at 1, 12 and 24 months, with 5–10 additional YWSS selected at each data collection round based on age and different levels of engagement with a range of DREAMS activities and services. In the end, we prospectively followed 16 respondents, of whom 12 were interviewed twice and four interviewed three times. The others were lost to follow-up either because they had moved out of the area, or they no longer wished to participate in the study. A further 27 YWSS were interviewed once, nine each at baseline, midline and endline (Fig. 1). Two female social scientists from the evaluation team with experience in qualitative research conducted the interviews in Shona or Ndebele at a location convenient to respondents. Interviewers contacted respondents through DREAMS programme staff, who explained the aims of the research and referred interested YWSS to fieldwork team. Interviews were transcribed and translated into English. Interviews followed a topic guide (provided as supplementary file) that led respondents chronologically through their experiences of learning about DREAMS, deciding to enrol, and subsequent activities including how and when they terminated their involvement. For the follow-up interviews, additional questions were tailored to each of the 16 respondents based on information they previously provided.

**Table 1:** Activities in DREAMS intervention and comparison arms

Intervention arm	Standard care
• Sisters’ clinical services • Peer educators • Community mobilisation with Activity Pack • Paralegal advice • HIV testing • Referrals to PSI / govt clinics for ART • Referrals to PSI clinics for PrEP • Referrals to 6 other DREAMS partners • Case care workers (lay child protection) • Accompanying/following up referrals • Young peer educators • Outreach workers • Adherence Sisters • Microplanning • WhatsApp discussion groups	• Sisters’ clinical services • Peer educators • Community mobilisation • Paralegal advice • HIV testing • Referrals to govt clinics for ART

During analysis, we applied the Theoretical Domains Framework (TDF) proposed by Michie et al. [16] for structuring identified facilitators and barriers of participation in DREAMS according to domains originally identified to help guide design and implementation of theory-driven interventions. The TDF brings together 33 theories of behaviour and behaviour change assembled into 12 original domains later expanded to 14 [16, 17] categorised into cognitive, social and contextual determinants of behaviour [18]. TDF domains most relevant to how DREAMS hoped to reduce YWSS’ vulnerability, for example, include knowledge; skills; beliefs about capabilities; motivation, intentions and goals; context and resources; and social influences [16]. We found that both facilitators and barriers to DREAMS fit several TDF domains, providing useful conceptual architecture to our understanding of why and how the DREAMS programme was able to engage those it targeted.

Following data familiarisation, we defined a coding frame that mapped emerging themes onto selected TDF domains (Table 2). Two researchers independently coded one transcript to test the framework and refine codes to improve inter-researcher consistency. They subsequently independently coded transcripts using NVIVO, grouping codes into *barriers* and *facilitators* in keeping with the research questions. We used NVIVO Case Classifications to collate demographic details about the YWSS interviewed in the study. We explored how DREAMS interventions were experienced by individual YWSS and examined factors associated with facilitating or hindering DREAMS intervention delivery from the perspective of YWSS involved at different time points. All names used in this paper are pseudonym.

**Table 2:** Framing definitions and constructs using the Theoretical Domains Framework to analyse barriers and facilitators

Domain and definitions	Constructs to frame barriers and facilitators
**Knowledge:** An understanding of the existence of the DREAMS interventions	Knowledge about DREAMS opportunities Procedural knowledge about enrolment into DREAMS
**Skills:** Training or proficiency obtained through practice/DREAMS	Increased education and skills acquired through DREAMS Practice and application of skills
**Beliefs about capabilities:** Realisation about own capabilities, talents and ability to translate new information and skills to constructive use	Self-efficacy Self-confidence / Self-esteem Perceived competence Empowerment
**Motivation, intentions and goals:** Mental perceptions about the outcomes that YWSS believe they can achieve through conscious behaviours that facilitate goals achievement	Intrinsic ambitions or goals Perceived attitudes, beliefs or intention of peers Expectations of programme participation
**Context and resources:** Any circumstance of YWSS’ context that hamper or facilitates the development of skills and capabilities, confidence, independence, optimism, perseverance, social competence and adaptive behaviour or to attain goals	Available financial or material resources Expenses incurred by participation Opportunity costs (childcare, familial responsibilities)
**Social influences:** The interpersonal actions that lead to a change of thoughts, feelings, or attitudes to participating in DREAMS	Social support: new friendships and social networks Mentorship/ approval from adults: positive messages from teachers/ family members Social comparisons/ personal or group social identity Social pressure—power/hierarchy resulting in stigma and discrimination DREAMS implementation dynamics -supervision and follow up of participant, community champions, enrolment, information dissemination, support structures Conflict—competing demands, conflicting roles, intergroup/family conflict and alienation

Ethical approval was granted by the Medical Research Council of Zimbabwe (MRCZ/A/2085) and the London School of Hygiene and Tropical Medicine (Ref 11,835), the UK. Written informed consent from participants were obtained before enrolment.

## Results

As shown in Table 3, 13 of the 43 participants were aged 18–19 years. Twenty-eight participants had lost one or both parents and half of these [14] had responsibilities caring for younger siblings. Seven of the 43 respondents were living with HIV. Among the 36 HIV negative participants, 29 were offered PrEP, 13 initiated PrEP and 7 continued taking it through the end of the study. 30 of the 43 participants were offered social protection programmes but not all programmes were of interest to all the participants. They participated in the programmes that were more appealing and relevant to them. All 43 women reported accessing the Sister’s clinical services with 18 reporting referral to DREAMS vocational skills training by Sisters (Table 3).

**Table 3:** Socio demographic profiles of participants

	**Variable**	**Number (*N* = 43)**	**Percentage (%)**
**1**	**Age in years**		
1.1	18–19	13	30%
1.2	20–25	30	70%
**2**	**Orphanhood**		
2.1	Lost one or both parents	28	65%
2.2	Has both parents	15	35%
**3**	**School status**		
3.1	In School	9	30%
3.2	Drop out	16	37%
3.3	Never went to school	1	2%
3.4	Out of school	17	40%
**4**	**HIV status**		
4.1	HIV Positive	7	16%
4.2	HIV Negative	36	84%
**5**	**Treatment status**		
5.1	Proportion of HIV + on ART	7	100%
5.2	Proportion of HIV- on PrEP	8	22%
**6**	**Household head**		
6.1	Household head	18	42%
**7**	**Teenage Pregnancy**		
7.1	Had chid(ren) before age 20	17	40%
**8**	**DREAMS Services offered**		
8.1	Sisters Clinic Service	43	100%
8.2	Vocational Training	18	42%
8.3	Second chance Education	12	28%

We examined the data for differences between the two sites. Main thematic findings did not vary, although some details about implementation of activities or services within individual clinics were site-specific. We focus on the broader determinants of YWSS’ entry and participation in DREAMS interventions, and present data by themes emerging across the whole sample. Reflecting the aims of the study we present YWSS’s experiences of DREAMS organised by key facilitators and barriers to uptake. We identified two main types of facilitators (Desire to escape poverty; Social support and friendships) and two of barriers (Financial constraints; Stigma and discrimination). We describe each of these in turn, alluding to relevant TDF domains (Table 2), which are addressed in the discussion that considers how interactions between these domains shape YWSS’ ability to take up and participate in DREAMS services.

### Facilitators to accessing and benefitting from DREAMS

The desire to escape poverty was the most influential facilitator for both starting and remaining engaged with DREAMS. Almost all participants described adverse childhood experiences catalysing an avalanche of negative repercussions. These included losing one or more parents, dropping out of school, selling sex, physical and sexual violence, economic impairment and, in 7 cases, acquiring HIV infection during their adolescence. At the core of these stories was the appetite and desire to escape the causes and consequences of poverty. A common thread through these individual narratives was driven to achieve *"improved"* life circumstances.

First, YWSS perceived poverty as a temporary condition that they could escape by improving their chances of employment through vocational skills development and education. They expressed desire to do better than their families by starting businesses, becoming hoteliers and engineers and securing salaried jobs. Acquiring new skills was highlighted as the main benefit of the DREAMS programme, and YWSS’ motivations to take advantage of these new opportunities were based on the belief that education and skills translated directly into livelihoods and future security for themselves and their families. YWSS associated higher levels of education and training with higher incomes, trusting that those who manage to acquire skills will be less likely to remain poor.

… I see that I may end up living my whole life in poverty like my family, unless I get a course [vocational skills training]. This is why this [DREAMS] is very important to me. I think I will have a better life eventually when I finish my course… If I was educated, I would get a better job. …. " **Nkosi, 21, training in events management**.

I will now be able to go and look for a job in hotels and even head a hotel one day. Once I get a job, I will look after my siblings and hopefully get them to school… **Rudo, 18, training in tourism and hospitality**.

Though I am still a sex worker, I think things are now much better [life after up taking DREAMS]. I will make sure that I do well in school, get a job and then be able to support my child and siblings. … I believe … I won't remain poor afterwards, no-way! **Lebo, 19, enrolled in School**.

Once enrolled in DREAMS, one of the main benefits reported by YWSS was the development of new friendships. The education and training courses exposed YWSS to different social spaces and networks, creating an environment that reinforced their intentions to succeed and strengthened self-belief in their ability to do so. For example, before the DREAMS intervention, Rudo felt lonely, depressed, lacked confidence and was afraid. When she started DREAMS, new friendships with girls in similar life circumstances encouraged her to remain engaged, reduced feelings of hopelessness, and bolstered her enthusiasm for participating more fully in vocational training.

I started feeling more powerful and alive after being friends with Kudzi [psuedonym]. We have amazing projects that we want to do together, and we are always encouraging each other. She is so clever, and I believe she will only invite a big harvest [success], not drought [poverty] in my life. **Rudo, 18, training in tourism and hospitality**.

Such support thrived where young women felt able to disclose their involvement in sex work to new friends, who became aware of their past struggles but nonetheless supported them in their transformations.

.. they [new friends] remind us to always focus on school and stop sex work especially Priscilla [pseudonym], who is always helping me. We lodge at the same house, and she encourages me to join her study [group] and tells me to stop being naughty [sex work]. **Deborah, 18, enrolled in School**.

Inhabiting new social networks helped some YWSS stay in the intervention long enough to perceive tangible benefits. Continuing Rudo’s story, her perseverance led to her seeing academic success, which further contributed to her increasing self-efficacy.

Soon after joining, I realised I actually had the capacity to do this [School]. I stopped self-pitying when others admired me for passing my first exam…I’m now more confident. **Rudo, 18, training in tourism and hospitality**.

In addition to peer support, some YWSS felt the DREAMS programme offered a welcoming environment. Often identified and denigrated for their involvement in selling sex, those YWSS who remained in DREAMS reported that they perceived a change in how they were approached, crediting specific teachers with changing their view of school. Having previously experienced stigma and poor treatment for selling sex, Bongani considered dropping out from her DREAMS-funded school course until a teacher intervened.

My teacher …. discouraged people [other students] from writing on the noticeboard about seeing someone in a compromising position over the weekend and so on [in sex work]. That was a turning point in my life and had high hopes to continue with school at a time I was almost giving up. **Bongani, 24, enrolled in School**.

Encouragement from other adult authority figures could also bolster YWSS’ confidence and intention to remain active in DREAMS. For example, Nthabiseng experienced bullying by other girls as a result of her YWSS identify. Kind words from her aunt maintained her motivation to remain in school.

I usually tell my aunt what they [other school pupils] have been saying [at school] and she advises me to keep focused [with school]. … She advises me not to pay attention to their destructive words. … You know, these [other school pupils] say a lot of hurtful words but I have learned to remain focused on my studies. This helps me and gives me strength and confidence to push my goals. **Nthabiseng, 20, enrolled in school**.

Finally, we found that some participants, like Rumbi, saw the intervention as an opportunity to challenge normative gender stereotypes. The programme’s focus on ensuring girls and young women have access to equitable opportunities inspired her to enter an occupation traditionally deemed “male”.

What made me study motor mechanics (DREAMS Vocational skills training) is that men look down upon women, and it makes women remain in poverty because they don’t do work that pays more, so I decided to venture into engineering. I want to be an Engineer and get out of this [poverty]. There is nothing that will stop me from going under a car to fix it if a man also does the same. This is very good for my confidence and how people now look at me. I feel respected and it helps me to respect myself even more…**Rumbi, 22, training in motor mechanics**.

Due to our purposive sampling that over-represented YWSS who engaged with DREAMS’ social protection component, we identified facilitators that proved sufficient to attract and retain a small proportion of all those eligible. Yet even DREAMS participants experienced difficulties in initiating or sustaining their involvement. We now turn to barriers to DREAMS participation.

### Barriers to accessing and benefitting from DREAMS

Although there were some differences between barriers to *uptake* and those to *retention* in DREAMS, financial constraints affected both. Opportunity costs and expenses incurred through participation in DREAMS education opportunities were most commonly mentioned as dissuading YWSS. Stigma and discrimination appeared to deter longer term engagement rather than initial enrolment into DREAMS.

The 15 interview respondents who either never enrolled or withdrew from DREAMS social protection described a range of challenges. Logistical barriers affected many. For example, participants described having no means to travel to the place of enrolment or not having a phone or credit to call the DREAMS contact person for enrolment.

I never attended school. We never had the money and I had to work to help my crippled mother then. So, when DREAMS came, I had an opportunity to go to school but I had no money to go to where they were taking people [enrolling]. When Ticha said he could help me to get there, the places were already full. **Teresa, 21, never enrolled**.

… when I wanted to register into vocational training, my friend told me that they only wanted individuals who are below twenty years old. **Jackie, 22, never enrolled**

…I lost the number of (DREAMS) the contact person when my phone got stolen. By the time I recovered it (phone), I decided to just forget about it because I thought it was too late. **Tanatswa, 19, never enrolled**.

I didn’t go because I had no money for transport ( enrolling site). **Piwayi, 23, never enrolled**.

Once receiving educational support, DREAMS paid for participants’ school fees but did not cover ancillary costs, leading to YWSS being unable to sustain the monetary demands of long-term involvement, e.g., school lunches, equipment and supplies. Lack of financial resources led some YWSS to withdraw due to shame and frustration. Inability to afford daily costs of attending school or vocational training highlighted the ways in which YWSS internalised their sense of worthlessness, or not being genuinely eligible for such programmes. For example, YWSS reported that not being able to eat during school lunchtimes exposed their poverty.

DREAMS just pay the school fees, but at times we have nothing to eat at school, nothing decent to wear, sanitary pads or stationery to use at school. Though DREAMS is doing a great job, we fail to complete our journeys because of these costs. **Fungai, 20, training in tourism and hospitality**.

I just don't go to class when I don't have the money. To be honest, I give an excuse that I'm feeling ill or something. We are too poor for this opportunity. These [DREAMS opportunities] work better for those with money. … It's just embarrassing and `heart-breaking at the same time. **Yolanda, 20, enrolled in school**.

Participants who had enrolled in the DREAMS food catering course also mentioned that they struggled to buy ingredients and needed uniforms such as aprons, and this resulted in them missing classes or dropping out (*N* = 5).

We sometimes don't go to school because we don't have … ingredients needed for the cooking class…Also, lack of smart phones is a problem become some of the lecturers send assignments on the WhatsApp group …. **Prisca, 19, training in hotel catering and management**.

Others could not afford the opportunity costs related to giving up other work such as selling sex or caring for children and other family members.

My plan was that maybe I should go on with school [through the DREAMS support] but sometimes I feel that maybe I should just look for something to do so that I can survive [food secure] and hopefully leave sex work, but it’s hard. **Fari, 20, enrolled in school**.

I can't leave her [younger sibling] alone … while I attend class [DREAMS school programme] … It makes it impossible to attend school even when the school fees are paid. This [childcare] happens especially in the farming season from October to March every year when they [parents] will not be around. **Thembi, 23, enrolled in School.**

Financial constraints also affected uptake of PrEP even though clinical services were popular, and nearly all DREAMS-eligible YWSS benefit from *Sister’s* outreach and clinics. Of 29 participants in this study who were offered PrEP, fewer than half initiated (*n* = 13) and only 7 continued taking PrEP. Reasons for discontinuation included inability to afford transports costs to the health facility, burden of having to take PrEP every day and stigma. Fari stopped taking the prophylaxis because she could not sustain transport costs when she needed refills.

I stopped taking it [PrEP] because I could not get transport money to get back for more … and my aunt …did not have money ….I know I’m at risk now, but what to do? **Fari, 20, enrolled in school.**

Respondents highlighted that social stigma around HIV undermined their ability to initiate and adhere to PrEP. YWSS experienced varied stigma including due to their involvement in sex work. While PrEP availability is not limited to sex workers in Zimbabwe, participants felt that being known to be starting PrEP "outs" them to their families as sex workers.

Okay, I did not want to take it up [PrEP] because if I get it and take it home, my family would like to know why I have PrEP. That’s like outing myself. Then they will suspect that I'm one [Sex worker]** Fundo, 22, training in ISAL.**

I refused to [take PrEP] because I was afraid that my mother would see the pills and she would ask me about them…it will be hard for me to stay at home because she will suspect that I'm doing sex work. **Prisca, 19, training in hotel catering and management.**

Stigma was also at the heart of most concerns about attending school. DREAMS participants were often identified as YWSS and denigrated by other pupils. Some contemplated dropping out of school yet again as a result of feeling mocked.

Some students in the same class with us [YWSS] mock and ridicule us saying that those on …scholarships [DREAMS] are sex workers. You will be thinking that you are fixing your life, but they mock you. So, I thought of quitting…" **Thuli, 19, enrolled in school.**

One of them [students] said …we want to tell the headmaster that we don't want to see maHure [prostitutes] in our class, referring to us. It's so discouraging. **Deborah, 18, enrolled in school.**

The term *"Hure"* in the Shona language is demeaning and carries a moralistic tone, negatively affecting the self-esteem required for uptake of DREAMS, as explained by Kuda;

Some will be asking why you have come to school, why did you leave your work of selling sex ("chihure") and make a scene in front of other people…. So, at times I feel so ashamed, and I would feel it's better not to attend school. **Kuda, 20, enrolled in school.**

The attitudes and behaviour of implementing staff could serve to dampen YWSS’ enthusiasm in the same way as it could encourage and motivate them. Unfortunately, fear of harassment by teachers was more frequently mentioned than their role as mentors.

Some of them [teachers] were saying that we are whores [sex workers]. They say that we are coming to school to disturb others [pupils] to learn, they [ teachers] ridicule us and also diss us and say that we won’t pass exams, we will always be beerhall people. **Deborah, 18, enrolled in school.**

The problem I have is the teachers stigmatize those getting fees from Umzingwane [DREAMS IP] as they automatically regard you as a prostitute or as if you are HIV positive. They even stigmatize you for the make-up that you wear. **Lebo, 19, enrolled in school.**

## Discussion

We describe facilitators and barriers faced by YWSS when engaging with the DREAMS interventions in Zimbabwe. Our data show that while there is widespread acceptance that adolescent girls’ and young women’s vulnerability needs to be addressed holistically through multisectoral approaches, in reality this is extremely challenging, particularly for young women at highest risk of HIV. Social and material deprivation are entrenched, and structural discrimination rife, contributing to the low rate of 9% of young women receiving integrated social services through DREAMS [14].

While the DREAMS package was comprehensive, providing a range of social and economic opportunities for YWSS, inequalities stemming from poverty, orphanhood, marital status, age and social capital influence one’s access to resources and the ability to exercise agency, including the ability to return to school even when the opportunity is there. The most vulnerable adolescent girls and young women selling sex have the least chances of accessing and benefiting from the available resources. They also have the least access to quality care during need and periods of risk, and this often results in a vicious cycle of vulnerability. This inequality has lasting repercussions for YWSS’ access to health services, educational opportunities, career and earning potential. Too many of them, continue to be left behind. The 2030 Agenda for sustainable development goals (SDG) offers an unprecedented opportunity to leave no one behind [19] and for AGYW to be heard, but inequalities continue to widen leaving many of them behind even in contexts of DREAMS. All SDGs processes and mechanisms take AGYWs’ needs, voices, and disparate experiences into account. Despite this, YWSS remain one of the most marginalized populations, facing the tripled discrimination of being young, female and sex worker. YWSS, who are orphaned, live in poverty, or head a household are even more likely to be left behind and invisible.

In our qualitative sample we were interested in comparing experiences of those who had taken up social protection opportunities with those who had not, selecting 43 YWSS who accessed *Sisters* with close to half (*n* = 18) reporting referral to DREAMS education or vocational training. Drawing on the TDF, we consider the strengths and weaknesses of DREAMS’ approach in terms of how participating YWSS’ engagement was shaped by determinants across key relevant domains and implications for future programming.

Our findings show that targeting highly vulnerable women such as YWSS with opportunities for new *knowledge and skills* through providing access to education and vocational training is not sufficient to ensure their ability to benefit from these. In this study, the YWSS did not always know how to navigate the enrolment process, and for many, financial constraints and structural discrimination that DREAMS aimed to address proved too great to overcome. Yet at the same time, YWSS were strongly driven by their desire to escape poverty and seek a better future with improved livelihood opportunities and reduced reliance on sex work.

Although DREAMS was designed as an HIV prevention and mitigation programme, we found that YWSS were more motivated by the social aspects of the programme, despite finding clinical services easier to access. Thus, social protection activities did reflect YWSS’ expressed *motivation, intention and goals*. Once enrolled, some YWSS successfully achieved a virtuous circle of positive reinforcement of their motivation levels through their DREAMS-supported school and vocational training. This was in large part due to exposure to new friendships, which provided new social networks and a supportive environment. Education helped YWSS forge friendships with peers who had similar ambitions to improve their lives as well as with girls and young women from less vulnerable backgrounds who accepted and encouraged them. This meant a change in the kinds of ***social influences*** to which they were exposed, which seemed conducive to helping overcome minor challenges and build commitment to the programme. Where teachers or other adults reinforced such support, YWSS’ ability to stay engaged with the programme increased.

Being able to see tangible positive change also enhanced YWSS *belief in their capabilities*, as demonstrated by the pride in which respondents described doing well academically or challenging gender norms by selecting “male” vocations. Self-esteem and self-efficacy could grow over time, and several YWSS indicated hopes to continue to gain additional qualifications and/or attend university.

The longitudinal analytical approach had its strengths; it allowed researchers to analyse change over time, carefully attributing participant behaviour to the DREAMS intervention. Additionally, the approach eliminated recall bias allowing researchers to make true conclusions about participants’ experiences engaging with the programme [20]. Participants who were interviewed multiple times were more sustained in the social protection program.

Yet individual attributes or experiences are unlikely to be adequate for creating an enabling environment for sustained participation. For example, those YWSS who did not develop new friendships or have broader social support from the outset of their involvement in DREAMS were more likely to struggle to overcome the significant barriers to their engagement. Ultimately, local *context and resources* shaped the ability of YWSS to join and remain in DREAMS programmes. Entrenched poverty and pervasive stigma of sex work combined to create insurmountable constraints for many eligible YWSS. Some could not afford the time to attend school or trainings due to their familial responsibilities, including meeting basic needs by earning money by selling sex. For others, the fact that “free” opportunities carried other costs such as transport, meals out, uniforms or ingredients meant they could not fully take advantage of them. Finally, the disappointment and shame of experiencing discrimination from fellow pupils and/or teachers reinforced existing doubts and increased chances that YWSS would drop out.

Our findings reflect experience in other settings where programmes for vulnerable populations struggle to overcome significant structural barriers, even as they offer services or opportunities in keeping with participants’ motivations and goals. Interventions addressing HIV and/or educational attainment for adolescents and young women, whether they are involved in selling sex or not, in contexts as diverse as Thailand, Malawi, India, and South Africa, have found that without addressing the wider context of social deprivation, it is difficult to reduce vulnerability to HIV and other poor health outcomes [21–24]. Furthermore, the context of Zimbabwe, which has seen recent political turmoil and economic deterioration, may have been particularly challenging for supporting individual-level change.

The fact that new friendships appeared to be an important facilitator of retention in the programme suggests one way forward for DREAMS and similar initiatives. While the social networks described in our study formed organically, proactively fostered community mobilisation might help develop such support [25, 26]. Community mobilisation activities are critical to developing strong relationships, networks, and collective strategies for overcoming social barriers and have successfully been used with sex worker communities to increase uptake of interventions [27–29]. Initiating the start-up of YWSS-led self‐help groups to build friendships, social cohesion, and trust could further increase the effectiveness of delivering DREAMS social protection, as for clinical services [30, 31].

The DREAMS intervention did not seem free of the stigma, experienced widely by YWSS from peers and even teachers. These findings suggest that interventions need to acknowledge and counteract deep-seated social stigma, including as it relates to poverty and gender norms. Previous research in Zimbabwe found that female sex workers living with HIV experienced greater discrimination for their involvement in sex work than for their HIV status [32]. The role of discrimination in discouraging YWSS’ engagement with health services is well documented [33–35]. In this study, we see that similar discrimination exists within the education sector and even programmes specifically targeting vulnerable groups. Focused policies, sensitisation and training for teachers, health workers, implementing partners, and others might help ensure that in future, more of the mentorship and encouragement from authority figures identified as facilitators of participation are integrated into programming and concomitant barriers reduced.

The rationale behind DREAMS was to provide multiple interventions to vulnerable girls and young women through a “layered” mechanism, whereby social and clinical services act synergistically to improve young women’s current circumstances and future life chances [15]. Our findings suggest that the provision of such a layered intervention should stretch even further. When YWSS do not know how much money they will have tomorrow, it becomes impossible to plan or continue engaging with the DREAMS programme. YWSS struggle to remain in school even if school fees are provided. When disaster strikes—whether in the form of an illness, or unavailability of a child minder—YWSS cope by disengaging from the programme so they can put more time in selling sex. Beyond offering school fees, broader support for participants’ livelihoods may be necessary, such as jobs, cash grants or microcredit. These might help YWSS remain in school by financing transport, uniforms and other needed school supplies – the circumstances that often restrict their ability to leave sex work and benefit from interventions [36].

For example, the SHAZ study (Shaping the Health of Adolescents in Zimbabwe) combined vocational training and health education, micro-grants and social support and compared to life-skills and health education alone. Although no difference in unintended pregnancies was observed, results revealed increased food security and income and reduced transactional and condomless sex [7]. An RCT of 25 primary schools in Zimbabwe showed that providing school fees and support costs such as bus fares to orphaned girls resulted in an 82% reduction in school drop-outs and marriage rates after two years [37].

## Conclusions

This study identified facilitators and barriers to participation in DREAMS among YWSS in Zimbabwe, which can help improve programmatic approaches to reduce HIV incidence among YWSS. Our study demonstrates that while poverty was perceived as an opportunity to improve life circumstances, it also constrained the ability to benefit fully from the DREAMS initiative. Multi-layered HIV prevention approaches such as DREAMS that seek to alter complex and longstanding social and economic deprivation address many of the challenges faced by YWSS but will only succeed if the underlying drivers of HIV risk among YWSS are also addressed.

## Supplementary Information

**Additional file 1.** Data collection instrument. Semi-Structured Interview Guide.

## Data Availability

Due to the criminalised nature of selling sex in Zimbabwe and the sensitivity of topics covered by interviews, the data will not be made publicly available. The authors will consider requests made to them directly for access to interview transcripts.

## Abbreviations

AGYW: Adolescent Girls and Young Women
DREAMS: Determined, Resilient, Empowered, AIDS-free, Mentored and Safe
ISAL: Internal Savings and Loans
PrEP: Pre-Exposure Prophylaxis
RCT: Randomised Controlled Trial
SDG: Sustainable Development Goals
TDF: Theoretical Domains Framework
YWSS: Young Women who Sell Sex
