# Supplementary material for: Facilitators and barriers to engaging with the DREAMS initiative among young women who sell sex aged 18–24 in Zimbabwe: a qualitative study
Source: BMC Womens Health. 2023 May 12;23:257. doi: 10.1186/s12905-023-02374-4 (PMC10182710; doi:10.1186/s12905-023-02374-4)
Supplement: Supplementary file 1 — Additional file 1. Data collection instrument. Semi-Structured Interview Guide. [file 12905_2023_2374_MOESM1_ESM.doc]

**
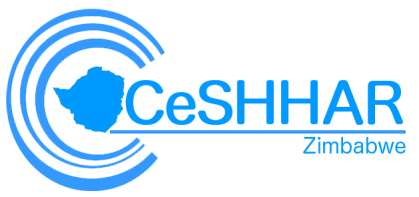
**

**The Centre for Sexual Health and HIV AIDS Research Zimbabwe (CeSHHAR Zimbabwe)**

**9 Monmouth Road, Avondale**

**Harare, Zimbabwe**

**Phone +263 4 332074**

21 Rowland Square, Milton Park,

Harare, Zimbabwe

________________________________________________________________________________

## Cross sectional qualitative interviews (6-monthly)

This interview is an opportunity to assess differences in experiences and perceptions of vulnerability in DREAMS intervention and comparison sites. Young women selling sex will be purposely selected for diversity across age, level of education, type of risk, high or low level of engagement in activities in interventions sites, experiences as Peer Educators, users and non-users of clinical services, those on and not on PrEP. ***Different women will be selected at each round***.

IDI respondents should undergo individual informed consented prior to the start of the interview according to GCP and as outlined in the SOP. Only young women who have provided written informed consent are eligible to be interviewed.

The IDI will be conducted in the language that the majority of women feel most comfortable in.

Introductory script

Hello, my name is XXX. We have selected you because you are a local young women in this area where we are conducting research. We would to interview you about your life experiences and the different kinds of programmes you’ve involved in or services that you use.

| **Outputs & Outcome thematic areas from DREAMS conceptual framework** | **Topic Area** | **Questions & Probes** |
| --- | --- | --- |
|  |  |  |
| Opening/ Background | Life circumstances | I’d like to get to know you. What can you tell me about yourself?   - Are you going to school? - How else do you spend your time? - What are your favourite activities? - Can you tell me about your family or the people with whom you live? - What are some of your concerns in life these days, or challenges that you face? - Please can you give me some examples? |
| Supportive Social Network & Sense of Community | Friendships  Support networks | So tell me about your friends?   - What kind of people are they and where do you know them from? - How many close friends would you say you have? - Can you tell me a little about them?   Where do you meet up with or hang out with your friends?  What sort of things do you do when you hang out with your friends?  If you have a problem or are worried about someone, which of these friends do you talk to?   - What kind of help do they provide? - Can you give an example? - Would you say that there is good trust between you and your friends? Why or why not?   What about other people in your life? Whom can you rely on?   - Family/ boyfriend/ relatives/ neighbours/ teachers…? - What kinds of support can you get from them? - Can you give an example of how someone has helped you or you have helped them? |
| Working with Others to improve life & work conditions | Peer relationships  Handling difficulties at work | Can you tell me a little bit about other young women like you? Maybe those whom you see when you are out selling sex. Do you consider other young women who sell sex to be your friends?   - Why or why not? - What does it depend on?   Tell me about your peers – what makes them like you, and what makes them different?   - Do you usually get along? - Why or why not? - What kinds of conflicts do you have?   Can you tell me about problems related to selling sex that you’ve experienced?  [Wait for response, then probe]   - Violence/difficult clients/ competition with others - What coping strategy do you use to overcome these challenges? - Will the others you work with help out? How?   Can you give me at least one example of a work-related problem?   - What happened? - How did others react? - Do you feel young women were supportive of each other in this situation? - Can you give me more examples? |
| Empowerment in Relationships  Using appropriate prevention strategies | Personal relationships  Condoms/Contraception | Can you tell me about any boyfriends that you have?   - What kinds of people are they? - What do you do when you’re together? - How do you feel about each other? - Can you describe some positive and negative parts of your relationship(s)?   Tell me a little more about your personal relationships. What makes you happy in them?   - Do you consider yourself to be in love? Why or why not? - What kinds of plans are you making for the future, if any?   Can you describe what it’s like when you’re together?   - For example, do you fight or argue a lot? - Do you feel that you can share personal problems or thoughts with your boyfriend? Please give examples of how you communicate and what sorts of issues you discuss. - Are you able to talk about what you do for work with your boyfriend(s)? How does he/ they respond? - How do you make decisions together as a couple, like about where to go or what to do, or how to spend your money.   What about in terms of sex?   - Can you describe how you communicate or take decisions about sex with your boyfriend(s), in terms of deciding whether or not to have sex and how often, or whether you will use contraception or condoms? - Do you talk about these things openly and easily? Can you describe how you made some of these decisions?   While we’re on the topic of sex, maybe you could tell me about using condoms or family planning – with your boyfriend(s) but also with your clients.   - What are your experiences of condoms and other contraception? - What do you like or dislike about condoms and other contraception? - What kinds of situations make it easy or difficult to use condoms or contraception? Can you give examples? - How is it different between your clients and your boyfriend? |
| Service uptake | Experience of DREAMS | So know I want to talk to you about your experience of DREAMS. First, can you tell me what the name “DREAMS” means to you? What do you think of?   - What services that DREAMS is offering young women have you heard about? - Which ones have you used? Can you list all the different parts of DREAMS that you have participated in? - Are there any others that you would *have liked* to participate in, but have not had the opportunity?   Please can you tell me the whole story of how you came to be involved in DREAMS? Start at the beginning when you first heard about the services, and then what happened?  So what do you like *best* about the parts of DREAMS you are involved in and why?  What do you like *least* and why?  Can you describe how you think the DREAMS services affect your life?   - Do you feel things are changing for you in any way as a result of your participation? Please give examples   If you have friends or peers that are also involved in DREAMS, how do you think it affects them?  What do you plan to do in the future with the DREAMS services.   - Do you plan to continue with all the parts you participate in? - Do you want to increase or decrease your involvement in any parts? Why or why not?   If you could change anything about the services you’re getting, what would you like to change? |
| Use of PrEP or ART  (If she has mentioned PrEP or ART as part of her experience of DREAMS, incorporate these questions there) | Prevention/ Treatment  [Ask about both PrEP and ART regardless of her status, but ask about her personal experience first – so if she says she is HIV- start with the PrEP question, and if she is HIV+ start with the ART question] | So now I’d like to talk about just 1 part of the DREAMS programme, specifically related to HIV services.  Can you tell me about any experience you have of HIV testing and what happened after you got the result?    Have you ever heard of PrEP, which is medication that someone who does not have HIV can take every day to reduce the chances of acquiring HIV infection?   - What do you know about it? - Do you know people who take PrEP? What do they say about it? - Has it been offered to you? Tell me about what happened and how you felt. - What did you decide? - [If on PrEP]: Please tell me about taking PrEP. What have been the benefits and challenges? Is it easy or difficult to take regularly? Please explain. - Have you told anyone you’re on PrEP? What do they say? [Give examples]   Have you ever heard of ART, which is medication that someone who is living with HIV can take every day to stay healthy?   - What do you know about it? - Do you know people who take ART? What do they say about it? - Has it been offered to you? Tell me about what happened and how you felt. - What did you decide? - [If on ART]: Please tell me about taking ART. What have been the benefits and challenges? Do you find it easy or difficult to take regularly? - Have you told anyone you’re on ART? What do they say? [Give examples] |
| Reduced perception of stigma, especially in use of services | Social stigma  Self-Stigma  Discrimination by service providers | Sometimes young women who sell sex get treated badly because of what they do. People say things or discriminate against them. Have you had these kinds of experiences?   - Can you describe them? - What kinds of people do you feel discriminate against you or other young women selling sex - How have you responded? - What kinds of people can you talk to about this kind of stigma or discrimation?   Are there any ways in which you have changed your behaviour because of being someone who sells sex? What I mean is, are there some things you would like to do or places you’d like to go, but you don’t because you feel embarrassed or nervous about how others would treat you? Can you give some examples?   - PROBE if respondent doesn’t have ideas of her own: Do you feel you act different at normal places where you go because of your work? (e.g. church, social events, school or other training opportunities, etc).   What about health services? Can you tell me about your interactions with different kinds of health providers and share any times you feel you’ve had difficulties because you sell sex?  Have you had any problems in the services provided through DREAMS? Can you give examples? |
